# Omalizumab and Dupilumab for the Treatment of Bullous Pemphigoid: A Systematic Review

**DOI:** 10.3390/jcm13164844

**Published:** 2024-08-16

**Authors:** Elena Granados-Betancort, Manuel Sánchez-Díaz, Daniel Muñoz-Barba, Salvador Arias-Santiago

**Affiliations:** 1 School of Medicine, University of Granada, 18071 Granada, Spain; 2 Skin Autoimmune Diseases Unit, Hospital Universitario Virgen de las Nieves, 18002 Granada, Spain; 3 Dermatology Department, Hospital Universitairo Virgen de las Nieves, Instituto de Investigación Biosanitaria IBS Granada, 18002 Granada, Spain; 4 School of Medicine, Dermatology Deparment, University of Granada, 18071 Granada, Spain

**Keywords:** bullous pemphigoid, omalizumab, dupilumab

## Abstract

**Background**: Bullous pemphigoid (BP) is an autoimmune disease characterized by the appearance of very pruritic subepidermal blisters. It appears mostly in the elderly and is associated with multiple comorbidities, which makes its management and treatment difficult. The purpose of this systematic review is to compile current information on published cases of BP treated with omalizumab (omalizumab) and dupilumab (dupilumab) in order to obtain information on clinical efficacy and safety data available. **Methods**: A literature search of all cases of BP treated with omalizumab/dupilumab published in the literature up to January 2024 was performed using the Pubmed database. After an exhaustive search, a total of 61 studies encompassing 886 patients met the inclusion criteria and were included in the review. **Results**: The majority of patients with BP treated with omalizumab/dupilumab presented a significant improvement in symptomatology, being very safe drugs with minimal side effects. The main limitation of the presented review is the quality of the included studies, most of them being case series or individual cases. The development of studies with a higher level of scientific evidence in the near future would be of great interest. **Conclusions**: Both omalizumab and dupilumab appear to be effective options for treating BP in patients refractory to other pharmacological therapies. They are drugs with a good safety profile and the adverse reactions associated with their use are infrequent and generally mild.

## 1. Introduction

Bullous pemphigoid (BP) is the most common autoimmune blistering disease in adults in developed countries [1]. It is characterized by the formation of autoantibodies against structural proteins of the dermal-epidermal junction and by the appearance of very itchy hives and subepidermal blisters [2].

Its incidence is about 0.2–3 new cases per 100,000 habitants [3], it appears more frequently in elderly patients (over 70 years of age) [4] and is associated with various comorbidities, such as neurological diseases, or other inflammatory diseases such as rheumatoid arthritis [5]. Probably in relation to the greater burden of comorbidities, as well as the clinical manifestations of the disease, the morbidity and mortality of patients with BP is 5 to 6 times higher compared to the general population adjusted for age and sex [1].

The pathogenesis of this disease is defined by an immunological component (IgG and IgE antibodies against hemidesmosomal proteins BP180 and BP230) and an inflammatory component (action of neutrophils and eosinophils that damage the dermo-epidermal junction). The deposit of antibodies in the basement membrane triggers an inflammatory response responsible for the clinical manifestations of the disease [1]. The ultimate cause is unknown, although exposure to certain drugs has been described as an etiological agent in some cases of BP [6].

The diagnosis of BP is based on the combination of clinical, histological, serological and immunofluorescence data. The suspected diagnosis must be clinical and requires a biopsy for histological and immunofluorescence study, as well as a serological evaluation [7,8].

Regarding current treatment of BP, it should be taken into account, that despite the availability of both topical and systemic treatments, such as corticosteroids and immunsupppresive drugs [9,10], the main limitation in the treatment of BP is the presence of side effects, which especially affect the typical patient group with BP, elderly patients and patients with multiple comorbidities.

Omalizumab is a humanized monoclonal antibody that selectively binds to immunoglobulin E. It is indicated for the treatment of severe allergic asthma, chronic spontaneous urticaria, and chronic rhysnosinusitis with nasal polyps. Various case reports have demonstrated the potential usefulness of omalizumab in BP, which could act by inhibiting the IgE-mediated inflammatory cascade, and is also a drug with an excellent safety profile [11]. On the other hand, Dupilumab is a drug that act son the α subunit of the interleukin 4 receptor (IL-4Rα) inhibiting IL-4 and IL-13 signaling. It is approved for the treatment of asthma, nodular prurigo, atopic dermatitis, and chronic rhinosinusitis with nasal polyposis. Currently, there are published cases in which a clinical improvement has been observed, with cessation of pruritus and reduction in blistering in patients with BP [12]. This improvement, as well as the absence of relevant adverse effects, makes dupilumab postulated as a treatment option for BP.

Given the recent evidence of the potential usefulness of omalizumab and dupilumab in the treatment of BP, as well as their excellent safety profile, it is of great interest to synthesize the available scientific evidence on their use in patients with BP, which is the objective of this systematic review. 

## 2. Materials and Methods

### 2.1. Study Design and Objectives

A systematic review was carried out including all the reports on BP treated with the biological drugs omalizumab and dupilumab, with the objective to analyze the common clinical characteristics, systematize the evolution of the disease, collect effectiveness data, as well as available safety data.

### 2.2. Search Strategy

A bibliographic search of all cases published in the literature up to January 2024 was performed using the Pubmed database. The search command used was: ((pemphigoid) OR (bullous pemphigoid)) AND ((omalizumab) OR (dupilumab)). The PRISMA 2020 guidelines for systematic reviews were followed when carrying out this work.

### 2.3. Inclusion and Exclusion Criteria

The search was limited to: (A) Publications on patients with a clinical diagnosis of BP regardless of severity and presentation treated with omalizumab and dupilumab. (B) Any type of epidemiological study (clinical trials, cohort studies, case-control studies, cross-sectional studies and clinical case presentations). (C) Articles written in English and Spanish. Therefore, the following were excluded: (A) Those publications that did not evaluate patients with a diagnosis of BP treated with omalizumab and/or dupilumab. (B) Clinical guidelines, protocols and conference summaries. (C) Publications written in a language other than English and Spanish.

### 2.4. Selection of Studies

A first search was carried out in which the titles and abstracts were reviewed by two researchers (EGB and MSD) of all the studies obtained when applying the search command. Of all those studies that met the inclusion and exclusion criteria, the full text was reviewed, as well as their bibliographic references in search of additional sources. Articles that raised doubts about their inclusion or exclusion were subject to discussion with a third researcher (SAS) until a consensus was reached. Articles considered relevant were included in the present analysis.

### 2.5. Research Questions

The present systematic review attempted to answer the following questions.

- What profile do patients with BP treated with biological drugs have?
- How effective are omalizumab and dupilumab in the treatment of BP?
- What is the safety and side effect profile of omalizumab and dupilumab in patients with BP?

### 2.6. Variables

To answer these questions, the variables evaluated were:

- Clinical and sociodemographic variables related to the characteristics of BP in patients treated with omalizumab/dupilumab, as well as the existence of comorbidities and other autoimmune diseases.
- Variables related to the therapeutic management carried out (treatment administered, dosage).
- Variables related to the effectiveness of treatment with omalizumab and dupilumab in BP. The rate of complete response of patients under treatment in the assessed studies was collected.
- Variables related to the safety of treatments.

### 2.7. Assessment of the Quality of the Scientific Evidence

The level of evidence of the studies included in the systematic review was evaluated according to the “Center for Evidence-Based Medicine” (CEBM). The levels of evidence were evaluated as follows:

- 1a: Evidence obtained from systematic reviews or meta-analysis of randomized controlled clinical trials.
- 1b: Evidence obtained from individual randomized controlled clinical trials.
- 2a: Evidence obtained from systematic reviews or meta-analysis of cohort studies.
- 2b: Evidence obtained from individual cohort studies.
- 3a: Evidence obtained from systematic reviews or meta-analysis of case-control studies.
- 3b: Evidence obtained from individual case-control studies.
- 4: Evidence obtained from case series.
- 5: Evidence obtained from expert opinions.

### 2.8. Statistical Analysis

Descriptive statistical techniques were used to evaluate the characteristics of the patients included in the evaluated publications. Continuous variables were expressed as mean and standard deviation. The qualitative variables were expressed based on their absolute and relative frequencies. Statistical analyzes were carried out with the JMP 9.0.1 program (SAS 105 Institute, Cary, NC, USA).

## 3. Results

**Figure 1:**
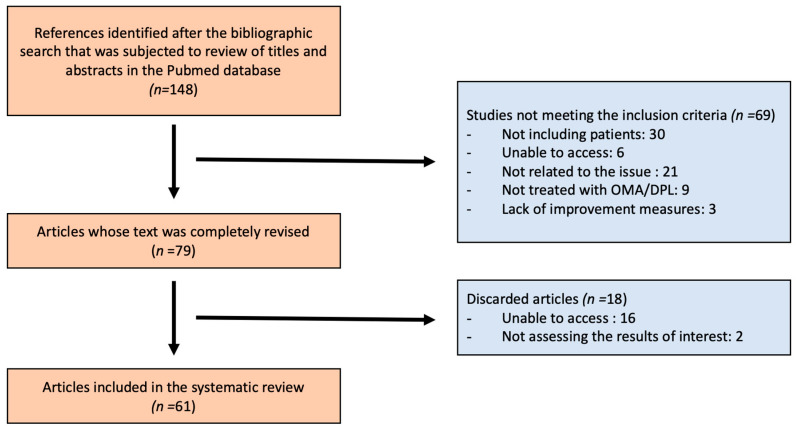
Search strategy. OMA (omalizumab); DPL (dupilumab).

After an initial search, 148 articles were found. After reviewing the titles and abstracts of each of them, 69 were discarded for not meeting the inclusion criteria. Therefore, 79 articles were completely reviewed, of which 18 articles were finally discarded since 16 of them were not completely accessible and 2 of them did not assess the impact of omalizumab/dupilumab on BP. Therefore, 61 articles were included in the systematic review that included 886 patients (363 treated with omalizumab and 523 treated with dupilumab) (Figure 1). All the information included in the studies can be seen in Appendix A and Appendix B.

### 3.1. Sociodemographic and Clinical Characteristics of Patients with BP Treated with Omalizumab and Dupilumab

A total of 363 patients with BP who were treated with omalizumab off-label were included in the review. The average age of the patients was 66.7 years. Of the studies that included the sex of the patients, the majority were women (44 vs. 36 men) although not all studies reviewed specified the age/sex of the patients. The majority of patients had multiple comorbidities associated with BP (diabetes mellitus, high blood pressure, osteoporosis, obesity, heart disease, chronic kidney disease, other associated autoimmune diseases). Furthermore, several cases developed BP as a consequence of treatment for other pathologies (such as dipeptidyl peptidase 4 inhibitors for diabetes treatment [13]; BP due to oncological treatment with anti-HER-2 drugs) [14]. The majority of patients had received treatment with several first-line drugs for BP prior to the initiation of treatment with omalizumab (corticosteroids, methotrexate), which had not achieved improvement in the disease.

Regarding patients with BP treated with dupilumab, a total of 523 off-label patients were included. The average age was 68.2 years, of which 59 were women and 174 men (although not all studies specified the age/sex of the patient). The majority of patients presented comorbidities typically associated with BP (cancer, immunosuppression, diabetes, heart failure, osteoporosis). In addition, several patients developed BP after starting treatment for another pathology they had (BP triggered by nivolumab for treatment of lung metastases due to melanoma [15], BP induced by pembrolizumab to treat cervical cancer [16]). Most of them were treated with other first-line drugs (corticosteroids or immunosupppresants) which did not achieve control of the disease.

### 3.2. Effectiveness of BP Treatment with Omalizumab/Dupilumab

Regarding the effectiveness of BP treatment with omalizumab and dupilumab, the following data were found (Table 1). Regarding treatment with omalizumab, the majority of patients with BP who were treated with 300 milligrams (mg) of omalizumab achieved complete remission of the disease (76.13%), achieving the disappearance of the characteristic skin lesions, as well as the pruritus. The time from the start of treatment to the improvement of the lesions varied among the cases presented, from two weeks to six months, with no recurrences after suspending omalizumab in most cases. Treatment duration was variable between case reports. In some cases, omalizumab was associated with rituximab, achieving remission of the disease more quickly [17]. Likewise, in many of these patients an improvement in the disease was observed, assessable by various scales such as the visual analogue scale (VAS). Along with this, the levels of IgE, eosinophils and antibodies also decreased anti-BP180, BP230.

Regarding patients with BP who were treated with dupilumab (600 mg induction + 300 mg maintenance), the majority (70.39%) achieved remission of the disease, with control of symptoms and resolution of blistering lesions, this being objective both by scales (EVA, bullous pemphigoid disease area index (BPDAI)) as well as laboratory data where many of them achieved a reduction in Th2 lymphocytes andantibodiesanti-BP180, BP230. The time to achieve improvement ranged from two weeks to six months, after which dupilumab was suspended, maintaining complete remission in most cases. The association of dupilumab with CTC achieved better BP control in certain cases [18,19].

### 3.3. Side Effects of Treatment with Omalizumab/Dupilumab

The majority of patients treated with omalizumab did not experience any adverse effects. Some adverse effects that were observed were: dermatitis at the injection site that resolved spontaneously, thrombocytopenia in two patients (one of them did not need to stop AOM and another of them who had multiple comorbidities died), intense pruritus that resolved by adding dupilumab and exacerbation of skin lesions in a patient who required discontinuation of omalizumab. The majority of patients tolerated the treatment adequately, with adverse effects being infrequent and mostly mild.

Most patients treated with dupilumab did not experience any adverse effects. Some presented eosinophilia (in two patients, it was resolved by adding immunosuppresive drugs), thrombosis on two occasions, dermatitis at the injection site that did not require suspending dupilumab, two patients developed pneumonia in relation to their comorbidities that did not require suspending dupilumab and were cured with antibiotics. As in the case of omalizumab, most of these adverse effects were mild and transient. 

## 4. Discussion

Omalizumab and dupilumab are two biological drugs widely used in dermatology for the treatment of pathologies such as chronic spontaneous urticaria and atopic dermatitis [1]. In light of the results of the present systematic review, they could be useful in the management of BP, given their effectiveness and safety data.

Regarding BP, both treatments, omalizumab and dupilumab could act on the pathogenesis of the disease. On the one hand, omalizumab acts as an anti-IgE drug, blocking anti-hemidesmosomal IgE antibodies which are involved in the development of the inflammatory reaction. On the other hand, dupilumab blocks IL-4 and IL-13 action, therefore inhibiting the Th2 pathway which is overexpressed in BP lesions [1]. 

The patients analyzed in the present systematic review who have received treatment with omalizumab and dupilumab are mostly elderly patients, with multiple comorbidities [4]. This study population resembles the patient profile commonly seen in real clinical practice [5], which adds value to the results obtained, facilitating the translation of the results of the review to medical practice. However, it should be taken into account that patients treated with omalizumab and dupilumab are mainly refractory to other treatments, which is why these drugs were administered off-label. The evaluation of the effectiveness and safety of the early treatment with biologic drugs for BP patients could be of interest to avoid the use of immunosuppressive drugs in patients with an increased comorbidity and mortality. 

Regarding effectiveness data, omalizumab and dupilumab could be considered effective drugs for BP, with complete response rates of 76.13% and 70.39% in the reviewed literature. These data are better than response rates seen in other studies for drugs such as methotrexate [20] or oral corticosteroids [21]. On the other hand, the dosage of omalizumab (300 mg subcutaneous every four weeks) and dupilumab (600 mg subcutaneous induction + 300 mg subcutaneous every one to two weeks) could favor greater adherence to treatment in elderly patients. Directly observed treatment (administered by nursing or qualified personnel) may even be useful [18,22]. Although exact complete response rates are not totally comparable due to the lack of standardized criteria among the included studies, future information on the clinical characteristics which could act as biomarkers of response to these drugs are necessary. The predominant implication of IgE antibodies or Th2 inflammatory response in each patient could make a difference in terms of clinical response.

On the other hand, it must be taken into account that the main limitation found in routine clinical practice for the treatment of BP is the comorbidity that patients present, in addition to their advanced age. This fact greatly limits the use of drugs that could be effective, such as systemic corticosteroids or immunosuppressive drugs, but which have an unfavorable side effect profile. Side effects found in the literature reviewed for omalizumab include injection site dermatitis, persistent pruritus, exacerbation of skin lesions, and thrombopenia. In the case of dupilumab, the most frequently described side effects are eosinophilia, infections and dermatitis at the injection site. In both cases, the profile of side effects is not very serious, which represents a comparative advantage with respect to the rest of the systemic treatments commonly used.

There are other biological drugs that could be useful for the treatment of BP, such as RTX an anti-CD20 drug. Although there is data on its effectiveness (with response rates of 70.5%) [22,23], Its side effect profile, which includes the possibility of serious infections and oncological processes, would a priori not make it optimal for the management of patients with BP with multiple comorbidities, which is why its data have not been the objective of this systematic review.

The main limitation of the review presented is the quality of the studies included in it, most of them being case series or individual cases. The development of studies with a higher level of scientific evidence in the near future would be of great interest for adequate knowledge of the degree of effectiveness of omalizumab and dupilumab in BP. Moreover, the lack of standardized criteria for defining clinical response in BP treatment could make it difficult to compare the effectiveness between both treatments. 

## 5. Conclusions

BP patients with treated with omalizumab and dupilumab show a profile of sociodemographic and clinical characteristics that could be comparable to that of patients with BP treated in routine clinical practice, although these are patients refractory to other systemic treatments, including corticosteroids and immunosupppresive agents. Both omalizumab and dupilumab appear to be effective options for the treatment of BP in patients refractory to other pharmacological therapies. Moreover, omalizumab and dupilumab are drugs with a good safety profile for use in patients with BP, with adverse reactions associated with their use being rare and generally mild.

## Figures and Tables

**Table 1:** Overview of data regarding omalizumab and dupilumab treatment for Bullous Pemphigoid.

	Omalizumab	Dupilumab
Number of patients treated	363	523
Approved indications for the drug	Severe allergic asthma, chronic spontaneous urticaria, chronic risnosinusitis with nasal polyps	Asthma, nodular prurigo, atopic dermatitis, chronic rhinosinusitis with nasal polyposis, eosinophilic esophagitis, hand dermatitis
Route of administration and dosage used in the studies	300 mg/450 mg/600 mg subcutaneously every 2–4 weeks	600 mg subcutaneously initially followed by 300 mg subcutaneously every 1–2 weeks
Efficacy data of omalizumab and dupilumab to treat BP	Complete response: 76.13%, improvement in BP between 2 weeks and 6 months after starting treatment	Complete response: 70.39%, healing achieved between 2 weeks and 6 months after starting treatment
Safety data	Less than 1% of patients presented adverse events, the most frequent were intense pruritus that resolved with dupilumab and dermatitis at the injection site that resolved spontaneously.	Less than 1% of patients presented adverse events, the most frequent were dermatitis at the injection site that resolved spontaneously and eosinophilia that resolved by adding IS.

## Data Availability

The original contributions presented in the study are included in the article, further inquiries can be directed to the corresponding author.

## Appendix A

**Table A1:** Summary of the Studies Including Patients Treated with Omalizumab.

Study	Type of Study	Number of Patients	Dose Used	Previous Treatments	Results Obtained	Side Effects	Observations	Level of Evidence
“Cao P et al. (2022)” [23]	Systematic review 75 studies included	211 patients with BP, 53 treated with omalizumab, 122 with RTX and 36 with dupilumab	NE, average treatment duration 6.6 months	CTC, MTX, MMF, AZA, CLP, CFF	Complete BP remission in 67.9% of patients (36/56) and partial remission in20.8% (11/53)	Recurrence 5.7% (3/53) Death due to thrombocytopenia(1.9%, 1/53)	The rest of the patients included in the study (122) were treated with RTX, giving a higher number of recurrences, AEs and mortality	2a
“Oren-Shabtai M et al. (2023)” [22]	Presentation of 9 cases	9, 3 of them treated with omalizumab, 7 with RTX and 1 with dupilumab, average age 60.4 years	300 mg every 4 weeks	CTC, BIO, RTX	78% clinical improvement, 55% complete remission at 3 months	None	omalizumab achieves greater IgE reduction. Multiple comorbidities: Parkinson’s, dementia, DM, HF, hypothyroidism, IR	4
“D’Aguanno K et al. (2022)” [24]	Systematic review of 22 articles	56	300 mg every 4 weeks. One patient received a single dose of 450 mg	CTC	87.5% respond to treatment at 13 weeks (55.4% complete remission, 32.1% partial)	None	NP	2a
“Seyed J et al. (2020)” [25]	Presentation of 1 case	1, M 70 years	300 mg every 4 weeks	CTC, DAP, MTX, MMF	Decrease in BP-100, EVA scale went from 9/10 to 2/10 after 2 months. Complete remission after 3 months	Persistent pruritus that was resolved by adding 600 mg dupilumab + 300 mg dupilumab	Complete remission with omalizumab + dupilumab after trying various treatments. Pcte with metabolic ds	4
“Vassallo C et al. (2022)” [26]	Retrospective study of 222 patients	222, 5 BP-dependent CTC patients with IC were selected for other treatments. Average age: 77.4 years. 3M, 2F	300 mg every 4 weeks, treatment duration: average 9.2 months	CTC, IS, AH	Resolution of skin lesions in all patients and reduction of pruritus. Reduction of IgE and anti-BP180, BP230 and eosinophils	NE	Patients with several comorbidities: DM2, hepatitis, hip replacement, vitiligo, osteoporosis	4
“Kremer N et al. (2019)” [11]	Systematic review of 35 publications	84 PCs (62 receive RTX and 22 omalizumab)	NE	CTC	Complete remission 85% with RTX and 84% with omalizumab	24% in treatment with RTX and 20% with omalizumab	Fewer recurrences with RTX than with omalizumab	2a
“Velin M et al. (2022)” [27]	Retrospective study	112 (19 met inclusion criteria), mean age 76 years	300 mg every 4 weeks SB, average of 9 months of treatment	CTC, MTX	60% complete remission, 20% partial remission	One case with poor skin tolerance (burning sensation) for treatment with dupilumab (lasted 1 month with treatment)	Of the 19 patients, 12 received MTX, 7 omalizumab and 8 dupilumab	4
“Kwon IJ et al. (2023)” [17]	Retrospective study	49 (25 RTX only, 17 RTX + omalizumab)	13 patients: 300 mg omalizumab twice (every 4 weeks) and 4 patients 300 mg omalizumab once	CTC, RTX	RTX + omalizumab improvement in 15 days vs. 67.5 days if only RTX. Control only RTX 92% vs. RTX + omalizumab 100%	None	0% mortality RTX + omalizumab, 16% if RTX monotherapy	4
“Yu KK et al. (2022)” [28]	Open, uncontrolled study	6, F, average age 72.8 years	300 mg every 2–4 weeks (6 cycles)	CTC, AZA, MC	Benefit in 5/6 patients with reduction in pruritus, eosinophils, blisters at 2 weeks	None	All failed previous treatment with CTC	4
“Seyed J et al. (2019)” [29]	Presentation of 2 cases	2, between 60–65 years	300 mg every 2 weeks for 1 month	CTC, AZA, TC	After 1 month of treatment with AOM, blisters and itching disappear	None	NP	4
“Alexandre M et al. (2022)” [30]	Retrospective study	13, 5M, 8F, average age 66 years	300 mg/450 mg/600 mg	CTC, MMF, DOX, MTX	Improvement in pruritus, hives, blisters, complete remission 85% at 3 months	2 pneumonias, 1 kidney failure in very frail elderly patients (no clear correlation with omalizumab use)	7/13 had mucosal involvement	4
“James T et al. (2019)” [31]	Presentation of 1 case	1, M 72 years	NE	CTC, IS, IVIg, RTX	Resolution of blisters, IgG decrease	NE	Multiple comorbidities: DM2, CKD grade IV	4
“Ewy S et al. (2019)” [32]	Presentation of 1 case	1, F 74 years	300 mg every 4 weeks	NE	Reduction of pain and skin lesions	Injection site dermatitis that resolved spontaneously within 2 days	NP	4
“Navarro-Triviño FJ et al. (2021)” [33]	Presentation of 1 case	1M, 70 years	300 mg subcutaneously every 3 weeks	CTC, AZA	Blisters disappear after 3 months	None	Analytical findings and refusal of patient to be treated with RTX due to PML risk	4
“Balakirski G et al. (2016)” [34]	Presentation of 2 cases	2, F 40 years old, M 63 years old	1. 300 mg every 3 weeks 2. 300 mg every 3 weeks	1. CTC, AZA 2. CTC	1 and 2: Pruritus improvement after 5 days of treatment with omalizumab	None	1. omalizumab was started at 300 mg every 4 weeks, but due to increased blisters, 300 mg was started every 3 weeks. 2. omalizumab was discontinued due to other health problems, reappearance of blisters, continued CTC	4
“Garrido PM et al. (2020)” [13]	Presentation of 1 case	1, F 76 years	300 mg every 4 weeks	CTC, DOX, AZA, IVIg	Pruritus disappears after 3 days, complete resolution of skin lesions after 2 months	None	BP triggered by treatment with DDP-4 (vildagliptin) for DM treatment	4
“From D et al. (2021)” [35]	Presentation of 6 cases	6 (4F, 2M), average age 64.5 years	300 mg every 4 weeks	NE, but CTC contraindicated due to comorbidities	NE	NE	Patients treated with AOM since due to their comorbidities they are IC other treatments	4
“Gönül MZ et al. (2016)” [36]	Presentation of 1 case	1, M 70 years	300 mg every 4 weeks, total of 11 doses	CTC, TC, DAP	Disappearance of blisters, IgE decrease a week after treatment	Thrombocytopenia that did not require discontinuation of omalizumab	NP	4
“Lonowski S et al. (2020)” [37]	Retrospective study	11, average age 78 years	10 treatments with 300 mg omalizumab every 4 weeks. One treatment with 375 mg every 2 weeks	CTC, RTX, AZA	6/11 complete answer 3/11 partial answer 2/11 no response	1 of them exacerbation of skin lesions that required discontinuation of omalizumab 1 of them had infection, died and had treatment with CTC 9/11 no adverse effects	The exacerbation of lesions with AOM was an AE that had not previously appeared and is the result of future research.	4
“Menzinger S et al. (2018)” [38]	Presentation of a case	1, F 76 years	300 mg every 4 weeks	CTC	Disappearance of the disease after 8 weeks of treatment, itching improves after 2 days	None	Multiple comorbidities (CKD dementia, ischemic heart disease…)	4
“Liu J et al. (2022)” [39]	Presentation of a case	1, M 76 years	300 mg every 4 weeks	AH, CTC	After 3 days itching improved, no new blisters appeared	NE	Multiple comorbidities that CI tto IS	4
“Yalcin AD et al. (2014)” [40]	Presentation of a case	1, M 28 years	300 mg, 13 doses in total	CTC, CFF	Complete remission	NE	Young person (28 years old)	4
“London VA et al. (2012)” [41]	Presentation of 1 case	1, F 70 years	300 mg every 4 weeks	CTC, AZA, MMF, CFF	Disappearance of blisters, decrease in anti BP180, disappearance of findings in IF	None	NP	4
“Barrios DM et al. (2021)” [14]	Retrospective study	34, 50% F mean age 67.5 a (9 of them due to BP) due to QT drug effects	NE	Combination with ipilimumab, atezolizumab, durvalumab	omalizumab improves pruritus and BP	2/9 did not respond to omalizumab)	Treatment with AOM for complications QT treatment in solid tumors	4
“Sinha S et al. (2020)” [42]	Presentation of a case	1, F 44 years	450 mg	CTC, RTX, AZA	Disappearance of blisters	NE	Obesity	4
“Dufour C et al. (2012)” [43]	Presentation of 1 case	1M 5 months	100 mg, every 2 weeks for 3 months	CTC, AZA	Reduction of urticarial lesions and blisters. Complete resolution after 10 months of treatment	NE	BP in a 5-month-old baby	4
“Fairley JA et al. (2009)” [44]	Presentation of a case	1, F 70 years	300 mg every 2 weeks	CTC, AZA, MC	Partial remission, decrease in eosinophils and anti BP180	NE	NP	4
“From A et al. (2021)” [45]	Presentation of 3 cases	1 of them with BP, M 65 years old	300 mg every 4 weeks	CTC, MMF	Complete remission after 3 months of treatment	NE	NP	4
“Chebani R et al. (2024)” [46]	Retrospective study	100, average age 77 years	300 mg	NE	Complete remission 77% at 3 months	NE	More significant improvement if high levels of anti BP180	4

AF: family history, HBsAg: hepatitis B surface antigen, AH: antihistamines, ATB: antibiotic, AZA: azathioprine, BIO: biological, BPDAI: bullous pemphigoid disease area index, CFF: cyclophosphamide, CH: colchicine, IC: contraindication, CLP: cyclosporine, CMV: cytomegalovirus, CTC: corticosteroids, DAP: dapsone, DM: diabetes mellitus, DOX: doxycycline, dupilumab dupilumab, CKD: chronic kidney disease, VAS: visual analog pain scale, F: woman, AF: atrial fibrillation, HTN: arterial hypertension, IC: heart failure, IDPP4: dipeptidyl peptidase 4 inhibitors, IF: immunofluorescence, Ig: immunoglobulins, IVIg: intravenous Ig, IM: immunomodulators, IR: renal failure, IS: immunosuppressants, PML: progressive multifocal leukoencephalopathy, M: male, MC: minocycline, MG: milligrams, MMF: mycophenolate mofetil, MTP: methylprednisolone, MTX: methotrexate, No.: number, NE: not specified, NP: not applicable, NT: nicotinamide, AOM: omalizumab, PA: bullous pemphigoid, PCTE: patient, QT: chemotherapy, PROM: premature rupture of membranes, RTX: rituximab, SB: subcutaneous, SD: syndrome, SEM: week, OS: week of gestation, TBC: tuberculosis, TC: tetracyclines, PET: pulmonary thromboembolism, TTO: treatment, HIV: human immunodeficiency virus.

## Appendix B

**Table A2:** Summary of the Studies Including Patients Treated with Dupilumab.

Study	Type of Study	Number of Patients	Dose Used	Previous Treatments	Results Obtained	Side Effects	Observations	Level of Evidence
“Cao P et al. (2022)” [23]	Systematic review 75 studies included	211 in total, 36 treated with dupilumab, 53 with omalizumab and 122 with RTX	NE	CTC, MTX, MMF, AZA, CLP, CFF	Total remission 66.7% (24/36) Partial remission 19.4% (7/36) at 4.5 months	Recurrence 5.6% (2/36). No adverse effects	The rest of the patients included in the study (122) were treated with RTX, giving a higher number of recurrences, AEs and mortality	2a
“Russo R et al. (2022)” [47]	Literary review of 9 articles	30 (16 M, 14 F), average age 69.85 years	NE	CTC, I.S.	Decrease in Th2 lymphocytes and improvement in pruritus	None, no interaction with other drugs	Some comorbidities: TB, melanoma, cancer, obesity, DM…	2a
“Zhao L et al. (2023)” [48]	Retrospective cohort study	146, average age 73 years, 86% M	300 mg every 2 weeks after an initial dose of 600 mg	NE	127 (87%) BP control in 1 month	Injection site injuries did not require suspending dupilumab	3 pctes pneumonia that improved with ATB without needing to suspend dupilumab (pneumonia associated with comorbidities)	2b4
“Zhang Y et al. (2021)” [49]	Retrospective study	24, average age 64.50 years (8 treatment with dupilumab + AZA + MTP and 16 AZA + MTP)	600 mg initially, followed by 300 mg weekly	MTP, AZA	Complete remission 62.5%, partial remission 12.5%	Eosinophilia, recurrence 12.5%	Add dupilumab to MTP + AZA + effective than without dupilumab	4
“Liang J et al. (2023)” [50]	Number of cases	9, average age 68 years	NE	CTC	Total remission: 74.6% Partial remission 11.1%	NE	NP	4
“Abdat R et al. (2020)” [12]	Case series from 5 academic centers	13, average age 76.8 years	NE	NE	Total remission: 53.8%, response to treatment 92.3%	None	NP	4
“Yan T et al. (2023)” [18]	Retrospective cohorts	40 (20 treated with dupilumab and another 20 with dupilumab + CTC)	600 mg initially followed by 300 mg weekly	CTC	Of the 20 treated only with dupilumab: 12 had complete remission, 8 had partial remission after 6 months of treatment.	NE	dupilumab improves AP symptoms, but fails to reduce BP180 levels	2b
“Oren-Shabtai M et al. (2023)” [22]	Series of 9 cases	9, 1 of them treated with dupilumab, 3 with omalizumab and 7 with RTX average age 60.4 years	600 mg initially, followed by 300 mg weekly	CTC, BIO, RTX	78% clinical improvement, 55% complete remission	None	NP	4
“Learned C et al. (2023)” [51]	Retrospective study	17 (10 M and 7 F), average age 72.2 years	300 mg weekly	MMF, DOX, CTC IVIg	14 patients had complete remission, 2 had partial remission and 1 had significant improvement.	None	The patients included had tried 4 lines of treatment prior to dupilumab	4
“Seyed J et al. (2020)” [25]	Description of a case	1, M 70 years	600 mg initially, followed by 300 mg weekly	CTC, DAP, MTX, MMF, omalizumab	Disappearance of pruritus, VAS scale 0/10, complete remission in AOM association	NE	Complete remission with omalizumab + dupilumab after trying various treatments. Pcte with metabolic ds	4
“Velin M et al. (2022)” [27]	Retrospective study	112 (19 met inclusion criteria)	300 mg every 2 weeks	CTC, MTX	60% complete remission, 20% partial remission	Only 1 percent in treatment with dupilumab skin burning sensation, only lasted 1 month with dupilumab	Of the 19 patients, 12 received MTX, 7 omalizumab and 8 dupilumab	4
“Hu L et al. (2023)” [52]	Retrospective study	11, average age 76 years, 4M, 7F	600 mg followed by 300 mg every 2 weeks	I.S., M.C.	In 2 weeks 10/11 patients control the disease	None	NP	4
“Qi W et al. (2023)” [53]	Compare 2 groups	27 (9 received MTP + dupilumab), 18 only MTP, mean age 72 years	NE	MTP	Improvement of the disease in patients treated with MTP + dupilumab	None with dupilumab	NP	4
“Klepper EM et al. (2021)” [54]	Case report	1, F 79 years	600 mg initially, followed by 300 mg weekly	CTC, DAP, DOX	After 1 month of treatment with dupilumab, 100% reduction in itching	NE	dupilumab indicated in people over 6 years of age	4
“Yang J et al. (2022)” [55]	Retrospective cohort study	40 (20 MTP only, 20 MTP + dupilumab)	600 mg initially, followed by 300 mg weekly	MTP	Greater control of BP, pruritus and quality of life in MTP + dupilumab	Eosinophilia, thrombosis in 2 patients (1 from each group), PE, gastritis, pneumonia, herpes zoster	NP	4
“Foerster Y et al. (2023)” [56]	Report of 3 patients, only 1 treatment with dupilumab	3 patients with BP + HIV-1, patients treated with dupilumab M aged 60 years	600 mg initially, then 300 mg every 2 weeks	CTC, AZA, DAP, DOX + antiretroviral treatment	Disappearance of itching and blisters	NE	Of the 3 exposed cases, only 1 was treated with dupilumab	4
“Zhang X et al. (2023)” [57]	Retrospective study	7	600 mg initially, followed by 300 mg weekly for 16 weeks	CTC, OMZ, tofacinib, CLP	6/7 complete remission 1/7 partial improvement	None	NP	4
“Sanfilippo E et al. (2023)” [58]	Report of 1 case	1, M 80 years	NE	CTC	Improvement of itching and disappearance of blisters	NE	Background: AF, HF, T2DM, HTN, prostate cancer, stroke	4
“Takamura S et al. (2022)” [59]	Presentation of 1 case	1, F 72 years	NE	NE	Improves pruritus, blisters and anti-BP180 negativity	NE	NP	4
“Wang Q et al. (2023)” [60]	Presentation of 1 case	1, M 60 years	600 mg initially, followed by 300 mg weekly	CTC, MTX	Improvement of itching in 3 days and blisters in 2 weeks, disappearance of Ig in 6 weeks	NE	Tto for 10 weeks after induction: CTC + dupilumab without AP recurrences	4
“Wang SH et al. (2023)” [61]	Number of cases	10 (7M, 3F) mean age 72.7	Initially 600 mg, then 300 mg every 2 weeks	MTP, MC, AH, IVIg	90% improvement in pruritus, complete remission 70%, average duration 8.3 weeks	Eosinophilia in 2 cases that was resolved with IS	Multiple comorbidities: DM, allergic rhinitis, osteoporosis, CMV infection, pneumocystis pneumonia	4
“Wang M et al. (2022)” [62]	Presentation of 2 cases	2	1. 300 mg dupilumab twice 2. 300 mg dupilumab twice	1. MTP + MTX 2. MTP	1. pruritus improvement in 2 weeks 2. lesion remission in 2 weeks	None	dupilumab prevents complications from other treatments such as RTX (infections and heart disease)	4
“Bruni M et al. (2022)” [15]	Case study	1, M 76 years	300 mg dupilumab	MTP, DOX	Complete remission in 6 months	NE	BP triggered by nivolumab for treatment of lung metastases due to melanoma	4
“Liu JH et al. (2023)” [63]	Case study	1, M 73 years old	600 mg subcutaneously, followed by 300 mg subcutaneously	DOX, CTC	Disappearance of PA lesions and psoriasis after 16 days of treatment with dupilumab. No relapses	NE	Effective treatment with dupilumab for psoriasis + BP	4
“Manzo Margiotta F et al. (2023)” [64]	Presentation of a case	1, M 74 years	600 mg followed by 300 mg every 2 weeks	CTC, DOX, NT, DAP	Resolution of blisters and itching after 16 weeks of treatment	None	NP	4
“Valenti M et al. (2022)” [65]	Case report	1	600 mg followed by 300 mg every 2 weeks	MTP, AZA, DAP, CH	After 3 months anti BP230 normal levels	NE	NP	4
“Savoldy MA et al. (2022)” [66]	Case study	1, M 78 years old	300 mg every 2 weeks	CTC, DOX, IS	Improvement after 6 weeks	None	AP triggered after COVID-19 vaccination	4
“Zhou AE et al. (2022)” [67]	Presentation of a case	1, F 17 years old	300 mg	CTC, RTX, IVIg	Complete resolution after 4 weeks, improvement after 2 weeks of starting dupilumab	NE, no relapses	Young woman (17 years old) with BP, not AF	4
“Pop SR et al. (2022)” [16]	Presentation of a case	1, F 59 years	300 mg dupilumab + CTC treatment	CTC, DOX, NT, DAP, MMF	Improvement of blisters	NE, CTC could be suspended without regrowth, leaving only dupilumab	BP induced by pembrolizumab for cervical cancer treatment.	4
“Riqueleme- Mc Loughlin et al. (2021)” [68]	Presentation of a case	1, F 37 years	600 mg at 30 weeks, followed by 300 mg at 2 weeks	CTC	Itching and blisters improve, fetus birth without incidents	PROM at 34.4 weeks, birth by cesarean section	Case of gestational AP + frequent in 2nd and 3rd trimester	4
“Zhang Y et al. (2021)” [69]	Presentation of a case	1, F 61 years	600 mg followed by 300 mg	CTC, AZA	Disappearance of itching after a month, no formation of new blisters	NE	NP	4
“Kaye A et al. (2018)” [70]	Presentation of a case	1, M 80 years	600 mg followed by 300 mg	CTC	Full resolution at 3 m and normalization levels BP180 and BP230	NE	TB infection and HBsAg + contraindicated immunosuppressive treatment	4
“Jendoubi F et al. (2022)” [71]	Presentation of a case	1, F 76 years	600 mg followed by 300 mg every 2 weeks	CTC	Complete resolution of pruritus and blisters, without recurrence after 6 months	None	Pcte with nodular pemphigus (BP variant)	4
“Fournier C et al. (2023)” [72]	Presentation of 3 cases	1, M 74 years old	600 mg followed by 300 mg	CTC	Complete remission	NE	Development of BP following treatment with nivolumab for melanoma	4
“Huand D et al. (2023)” [19]	Retrospective cohort study	36 patients, 20 receive MTP, 16 dupilumab +MTP, average age 71 years	600 mg of dupilumab followed by 300 mg athenext week	NE	Pruritus decrease and BPDAI scale improvement + effective with MTP + dupilumab at 2 weeks	2 cases dermatitis at injection site, 3 transient hyperglycemia, 4 hypereosinophilia	MTP + dupilumab group, MTP is suspended and 300 mg 2/wk dupilumab is continued as monotherapy.	4
“Chen J et al. (2023)” [73]	Presentation of 2 cases	2, M, 66 and 79 years	600 mg initially, followed by 300 mg weekly for 16 weeks	NE	Clinical improvement without relapses	One of them had erythema at the injection site, which resolved itself.	Comorbidities: DM, asthma, HTN	4

AF: family history, HBsAg: hepatitis B surface antigen, AH: antihistamines, ATB: antibiotic, AZA: azathioprine, BIO: biological, BPDAI: bullous pemphigoid disease area index, CFF: cyclophosphamide, CH: colchicine, IC: contraindication, CLP: cyclosporine, CMV: cytomegalovirus, CTC: corticosteroids, DAP: dapsone, DM: diabetes mellitus, DOX: doxycycline, dupilumab dupilumab, CKD: chronic kidney disease, VAS: visual analog pain scale, F: woman, AF: atrial fibrillation, HTN: arterial hypertension, IC: heart failure, IDPP4: dipeptidyl peptidase 4 inhibitors, IF: immunofluorescence, Ig: immunoglobulins, IVIg: intravenous Ig, IM: immunomodulators, IR: renal failure, IS: immunosuppressants, PML: progressive multifocal leukoencephalopathy, M: male, MC: minocycline, MG: milligrams, MMF: mycophenolate mofetil, MTP: methylprednisolone, MTX: methotrexate, No.: number, NE: not specified, NP: not applicable, NT: nicotinamide, AOM: omalizumab, BP: bullous pemphigoid, PCTE: patient, QT: chemotherapy, PROM: premature rupture of membranes, RTX: rituximab, SB: subcutaneous, SD: syndrome, SEM: week, OS: week of gestation, TBC: tuberculosis, TC: tetracyclines, PET: pulmonary thromboembolism, TTO: treatment, HIV: human immunodeficiency virus.
